# Tuning piezoelectric properties through epitaxy of La_2_Ti_2_O_7_ and related thin films

**DOI:** 10.1038/s41598-018-21009-5

**Published:** 2018-02-14

**Authors:** Tiffany C. Kaspar, Seungbum Hong, Mark E. Bowden, Tamas Varga, Pengfei Yan, Chongmin Wang, Steven R. Spurgeon, Ryan B. Comes, Pradeep Ramuhalli, Charles H. Henager

**Affiliations:** 1 Physical and Computational Sciences Directorate, Pacific Northwest National Laboratory, Richland, WA 99354 United States; 2 Materials Science Division, Argonne National Laboratory, Lemont, Illinois 60439 United States; 3 Department of Materials Science and Engineering, KAIST, Daejeon, 34141 Republic of Korea; 4 Environmental Molecular Sciences Laboratory, Pacific Northwest National Laboratory, Richland, WA 99354 United States; 5 National Security Directorate, Pacific Northwest National Laboratory, Richland, WA 99354 United States; 6 Energy and Environment Directorate, Pacific Northwest National Laboratory, Richland, WA 99354 United States; 7 Present Address: Department of Physics, Auburn University, Auburn, Alabama 36849 United States

## Abstract

Current piezoelectric sensors and actuators are limited to operating temperatures less than ~200 °C due to the low Curie temperature of the piezoelectric material. Strengthening the piezoelectric coupling of high-temperature piezoelectric materials, such as La_2_Ti_2_O_7_ (LTO), would allow sensors to operate across a broad temperature range. The crystalline orientation and piezoelectric coupling direction of LTO thin films can be controlled by epitaxial matching to SrTiO_3_(001), SrTiO_3_(110), and rutile TiO_2_(110) substrates via pulsed laser deposition. The structure and phase purity of the films are investigated by x-ray diffraction and scanning transmission electron microscopy. Piezoresponse force microscopy is used to measure the in-plane and out-of-plane piezoelectric coupling in the films. The strength of the out-of-plane piezoelectric coupling can be increased when the piezoelectric direction is rotated partially out-of-plane via epitaxy. The strongest out-of-plane coupling is observed for LTO/STO(001). Deposition on TiO_2_(110) results in epitaxial La_2/3_TiO_3_, an orthorhombic perovskite of interest as a microwave dielectric material and an ion conductor. La_2/3_TiO_3_ can be difficult to stabilize in bulk form, and epitaxial stabilization on TiO_2_(110) is a promising route to realize La_2/3_TiO_3_ for both fundamental studies and device applications. Overall, these results confirm that control of the crystalline orientation of epitaxial LTO-based materials can govern the resulting functional properties.

## Introduction

Piezoelectric materials that maintain their properties at very high temperatures are required for next generation piezoelectric sensors, actuators, transducers, and transformers utilized in nuclear^1,2^, automotive, and aerospace applications^3^. Traditional piezoelectric materials, such as Pb(Zr,Ti)O_3_ (PZT), possess a strong piezoelectric coefficient (d_33_) (200–400 pC/N for PZT), but Curie temperatures (*T*_*C*_’s) in the range of 300–365 °C limit their practical use to temperatures less than ~200 °C^2,4^. However, as the *T*_*C*_ of piezoelectric materials increases, the piezoelectric sensitivity tends to decrease^3,5^. For very high temperature applications, candidate piezoelectric materials with a high T_C_ include lithium niobate^4^ (*T*_*C*_ = 1150 °C, d_33_ = 6 pC/N), AlN^5^ (*T*_*C*_ = 1150 °C, d_33_ = 5.5 pC/N), and La_2_Ti_2_O_7_ (LTO)^6,7^ (*T*_*C*_ = 1500 °C, d_33_ = 16 pC/N). Among these, LTO possesses the highest *T*_*C*_ of any known piezoelectric material, and is stable in oxygen environments (in contrast to AlN^3^). Therefore, it is necessary to maximize the piezoelectric coupling of LTO for use in practical devices.

LTO crystallizes in the perovskite-like layered structure (PLS), which is related to the conventional perovskite structure (*AB*O_3_), with the insertion of an additional (110) oxygen plane after every fourth perovskite-like layer^8–10^. LTO is one end member of the perovskite family with the formula La_*n*_Ti_*n*_O_3*n*+2_ (*n* = 4, Ti^4+^ for LTO); the other end member is LaTiO_3_ (*n* = ∞, Ti^3+^, perovskite structure). Structures with 4 < n < ∞ possess an increasing number of perovskite layers between additional oxygen planes, and the appropriate mix of Ti^3+^ and Ti^4+^ to maintain charge neutrality. To distinguish La_2_Ti_2_O_7_ from the other members of this family, it is sometimes referred to as the “227” phase. In the PLS structure, each perovskite slab is offset from the underlying slab, resulting in a monoclinic structure (*a* = 13.019 Å, *b* = 5.547 Å, *c* = 7.811 Å, β = 98.28°)^6,11^. Distortions of the oxygen octahedra lead to ferroelectricity, with the ferroelectric polarization direction along the additional oxygen planes (along the *b* direction)^9^. This type of ferroelectric distortion is unusual in perovskites, since rotations of oxygen octahedra are typically antiferrodistortive^9^.

One way to increase the macroscopic piezoelectric response of LTO for use in sensors and devices is to align the piezoelectric direction across the material by synthesizing oriented^7^ or single^6^ crystals. One method to accomplish this is by deposition of epitaxial thin films of LTO on lattice-matched substrates. A similar approach was recently shown to enhance the piezoelectric response of epitaxial films of ferroelectric (Bi_0.5_Na_0.5_)TiO_3_–(Bi_0.5_K_0.5_)TiO_3_ with the (100) orientation^12^. Previously, epitaxial thin films of LTO have been deposited by molecular beam epitaxy (MBE)^13,14^, pulsed laser deposition (PLD)^15–17^, and sputtering^18^. SrTiO_3_ (STO) is the typical perovskite substrate choice (cubic perovskite, a = 3.905 Å). The best lattice match is found to be LTO(100) || STO(110), which places the additional oxygen planes parallel to the growth surface^13,14^. Epitaxial LTO $(\overline{2}10)$ films have also been obtained on STO(001) substrates; in this case, the monoclinic distortion of the LTO crystal structure results in a tilt of ~4.5° between LTO $[\overline{2}10]$ and STO[001]^16^. In this orientation, the additional oxygen planes, and thus the ferroelectric direction, are oriented approximately 45° out of the film plane.

We have identified rutile TiO_2_(110) as a potential substrate upon which to deposit epitaxial LTO(010) films, which has the potential to orient the piezoelectric direction out of the film plane. Rutile TiO_2_(110) possesses a reasonable lattice match to LTO in one direction, and a coincident lattice match in the perpendicular direction. In this paper, we present the crystallographic and piezoelectric properties of LTO epitaxial thin films deposited by PLD on STO(110), STO(001), and rutile TiO_2_(110) substrates.

## Results and Discussion

### LTO on STO(110)

**Figure 1:**
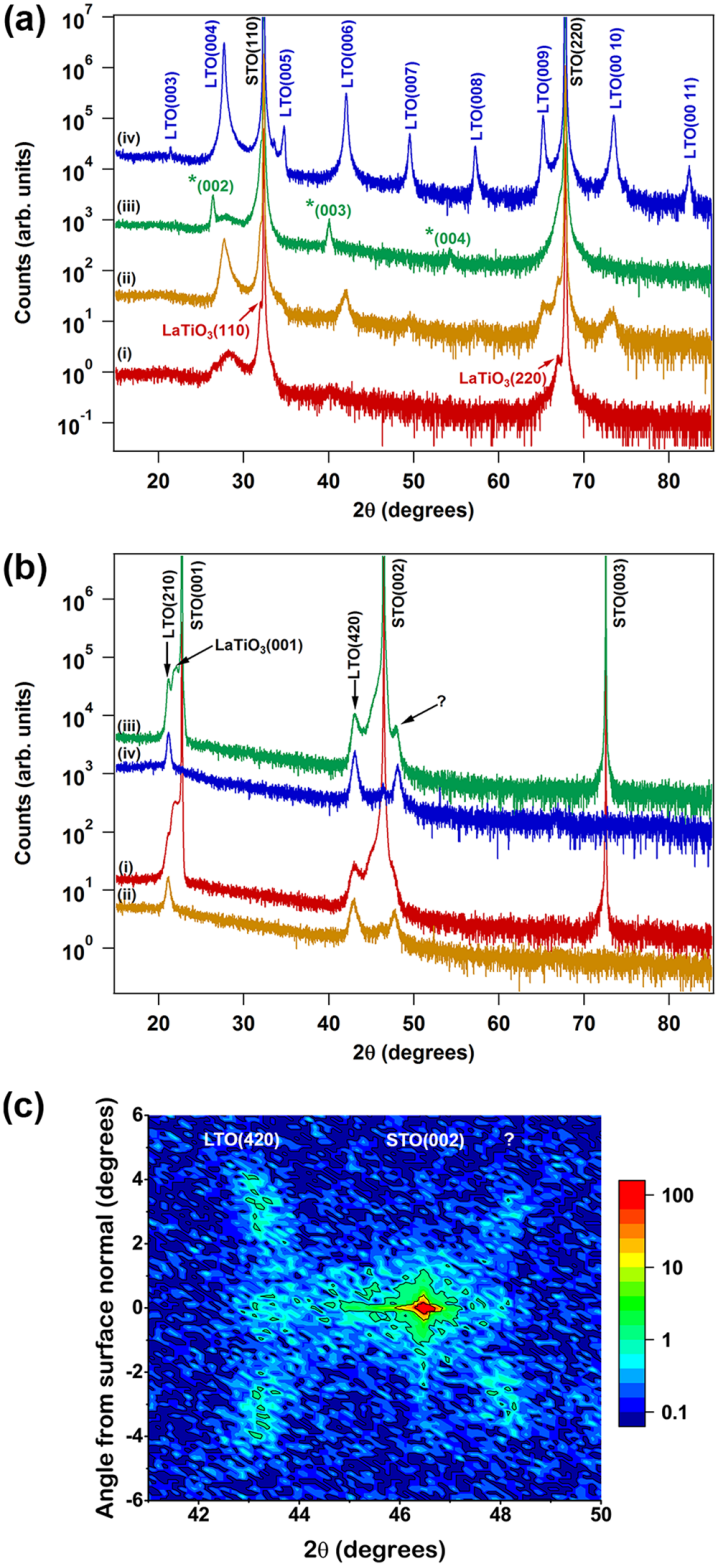
High resolution out-of-plane x-ray diffraction. (**a**) La_2_Ti_2_O_7_ deposited on STO(110) at 950 °C. Curve (i) as-deposited at 25 mTorr O_2_; (ii) film from (i) after annealing 1100 °C/4 h in air; (iii) as-deposited at 0.5 mTorr O_2_, phase marked with * is unidentified; (iv) film from (iii) after annealing 1100 °C/4 hrs in air, which has the desired PLS structure. (**b**) La_2_Ti_2_O_7_ deposited on STO(001) at 925 °C. Curve (i) as-deposited, measurement aligned to STO substrate; (ii) as-deposited, aligned to film peak at ~42°; (iii), (iv) annealed at 1100 °C/4 h in air, aligned to substrate and film, respectively. (**c**) Reciprocal space map of LTO(420), STO(002), and unknown (“?”) reflections from La_2_Ti_2_O_7_ deposited on STO(001).

As shown in Fig. 1(a), deposition of LTO on lattice-matched STO(110) did not result in a well-crystallized PLS phase for any deposition conditions (750–1000 °C, 0.5–120 mTorr O_2_). Many films produced an XRD pattern similar to curve (i) in Fig. 1(a), with a broad peak at ~25–30° 2θ. At best, the well-crystallized peaks observed in curve (iii) were obtained, although the broad peak was consistently present as well. Surprisingly, the sharp peaks in curve (iii) could not be indexed to any known La_*n*_Ti_*n*_O_3*n*+2_ phase. The peak spacing is consistent with a cubic lattice of repeat distance 6.75 Å. When films with this unidentified phase are annealed at 1100 °C for 4 h in air, the XRD pattern completely transforms to well-crystallized PLS with little or no evidence of the previous diffraction features, as shown in curve (iv). Annealing the poorly-crystallized film (curve (i)) under similar conditions produces a pattern (curve (ii)) which indicates partial crystallization in the PLS phase, but the broad peak widths and low intensities indicate that this film is poorly crystallized compared to the annealed film in curve (iv).

**Figure 2:**
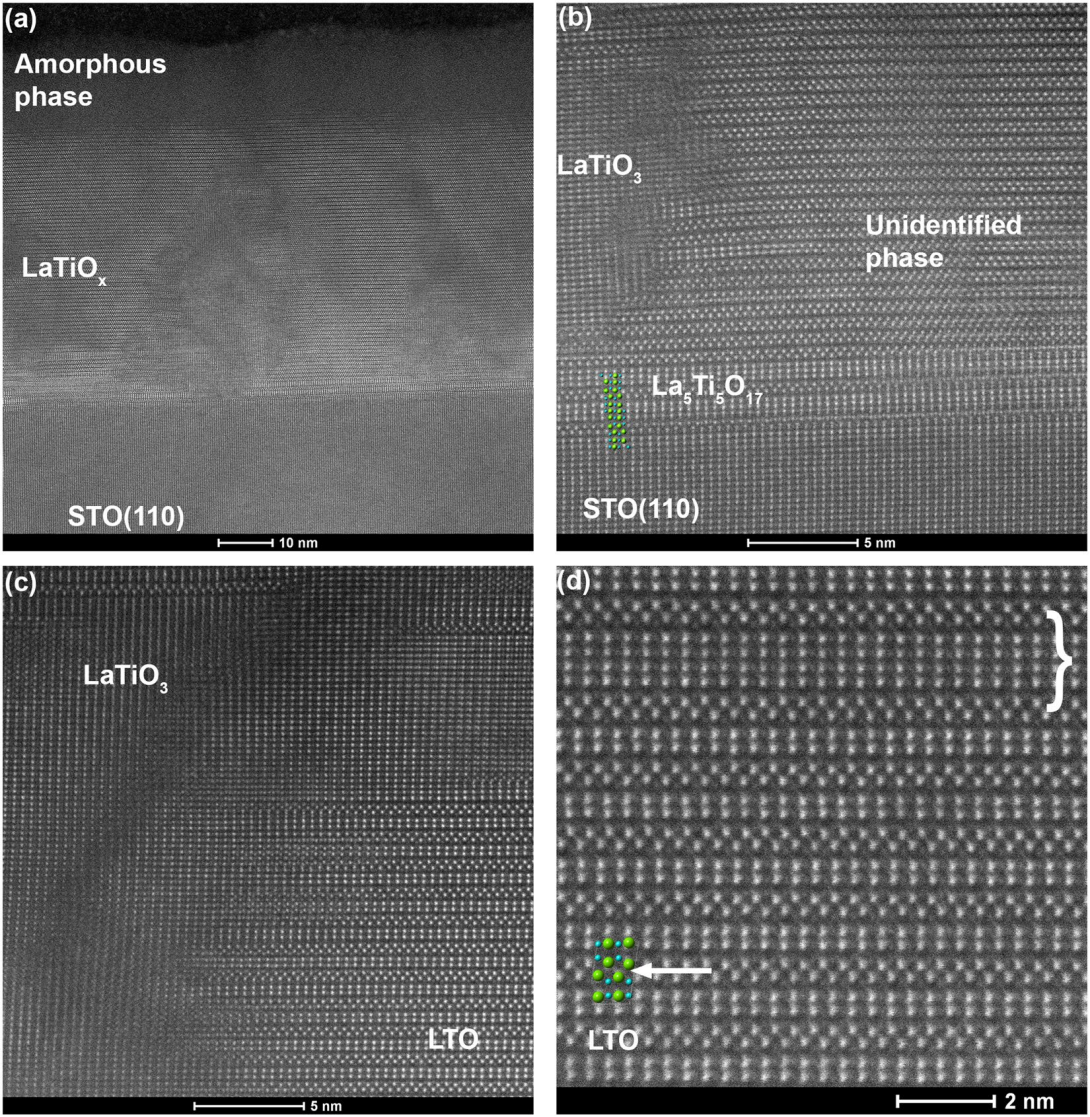
STEM-HAADF images of LTO/STO(110). (**a**), (**b**) As-deposited film. Regions of perovskite LaTiO_3_, La_5_Ti_5_O_17_ (including an overlay of the atomic positions in one unit cell), and an unidentified phase are indicated. (**c**), (**d**) LTO/STO(110) after annealing at 1100 °C/4 h in air. A region of perovskite LaTiO_3_ is indicated in (**c**). The PLS atomic positions in one unit cell is overlaid on the PLS lattice image in (**d**), and the position of the additional oxygen plane is indicated with an arrow. The curly bracket near the top of the image in (**d**) indicates a defected region in which 6 perovskite layers separate additional oxygen planes.

The XRD results for LTO/STO(110) are corroborated by STEM-HAADF imaging of the films before and after annealing. Figure 2(a) presents a cross-sectional STEM-HAADF image of LTO/STO(110) analogous to the XRD pattern of curve (iii) in Fig. 1(a). Although the film has a sharp interface with the substrate, significant disorder and phase separation are observed in the bulk of the film. A thick (10–15 nm) amorphous layer is present at the film surface. The high-resolution image in Fig. 2(b) reveals the complex microstructure of this film. On the left side of the image, a square lattice pattern is observed which appears similar to that of the STO(110) substrate; we tentatively assign this to perovskite LaTiO_3_. A disordered region separates this LaTiO_3_ phase from the well-crystallized phase that dominates the film. The repeat distance of this crystalline phase is ~7 Å, matching reasonably well (within the error of the STEM measurement) to the 6.75 Å repeat distance observed in the XRD pattern for the unidentified phase. At the LTO/STO interface, a unit cell of La_5_Ti_5_O_17_ (*n* = 5) has nucleated (Fig. 2(b)); La_5_Ti_5_O_17_ is similar to La_2_Ti_2_O_7_, but with five perovskite layers between additional oxygen planes^19^. It should be noted that the diffraction peaks observed in Fig. 1(a) do not correspond to the diffraction pattern of La_5_Ti_5_O_17_^20^. After annealing, the unidentified phase has transformed to a well-crystallized and stoichiometric PLS phase, as shown in Fig. 2(c) and (d). As expected from the XRD patterns, the additional oxygen planes lie parallel to the STO substrate, with LTO[001] // STO[110]. In addition to the PLS phase, regions of perovskite LaTiO_3_ still remain.

**Figure 3:**
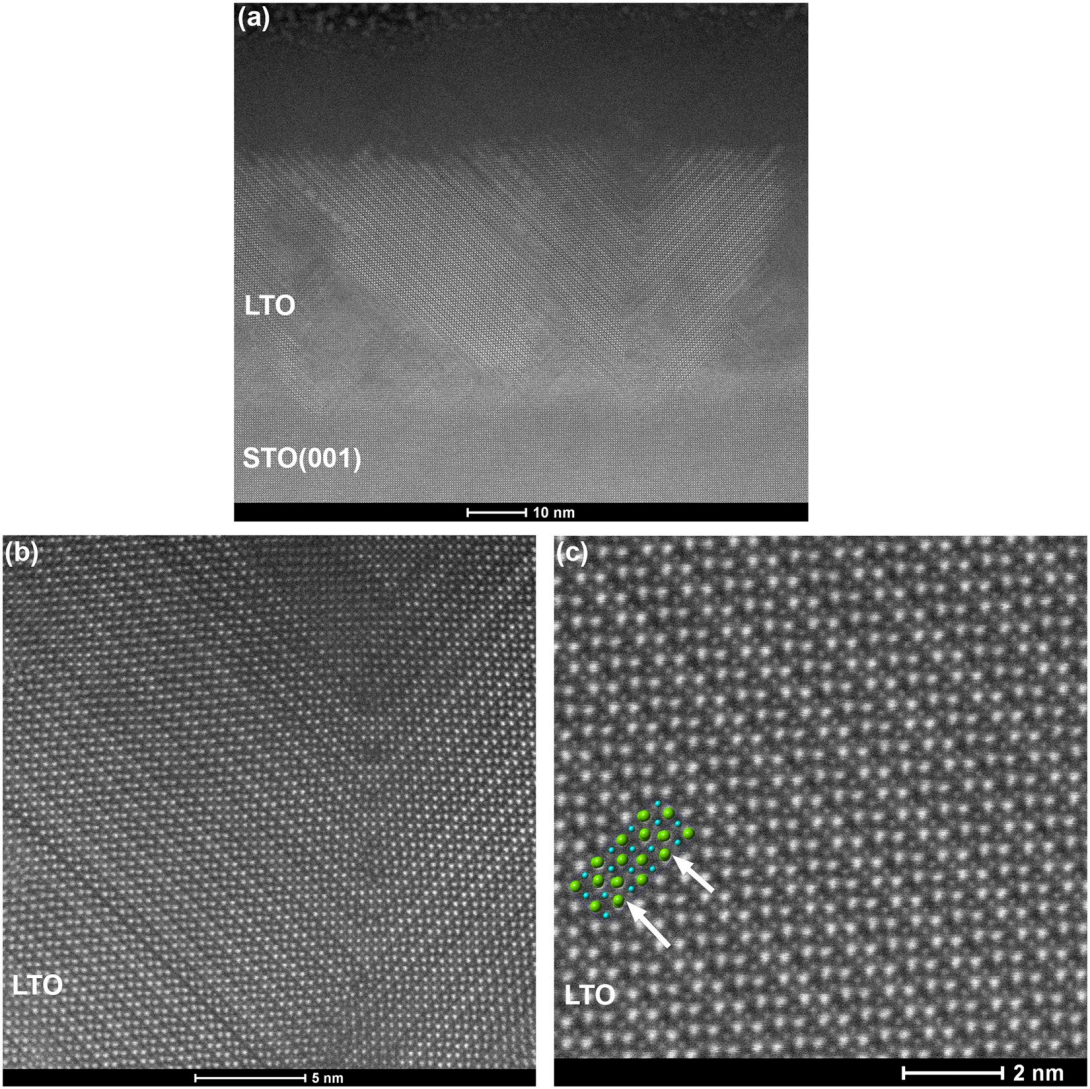
STEM-HAADF images of LTO/STO(001) after annealing at 1100 °C/4 h in air. Two PLS unit cells are overlayed on the PLS lattice image in (**c**), and the positions of the additional oxygen planes are indicated by arrows.

The behavior of La-Ti-O films deposited on STO(110) as a function of oxygen pressure during deposition is counterintuitive. Deposition at low oxygen pressure (0.5 mTorr) results in an unidentified phase; annealing in air recovers the PLS LTO phase. It might be expected that deposition at low oxygen pressure results in an oxygen-deficient phase, and increasing the oxygen pressure would result in the direct formation of PLS LTO. Instead, deposition with increased oxygen pressure results in poorly-ordered or nearly amorphous films. This points to the substantial energy required to form the PLS phase: in our PLD system, the relatively large distance between target and substrate (7.5 cm) means that an increase in background oxygen pressure significantly decreases the kinetic energy of species in the ablation plume, and this reduced kinetic energy is insufficient to form the PLS phase at the growth temperature employed (≥900 °C).

The unidentified phase observed in Fig. 1(b), curve (iii) and Fig. 2(b) closely resembles both the La_2_Ti_2_O_7_ and La_5_Ti_5_O_17_ crystal structures, and in fact appears to match the *A*_2_*B*_2_O_8_ layered structure^10^. Although “La_2_Ti_2_O_8_” cannot exist as a charge-neutral compound (only Ba*M*F_4_ with *M* = Mn, Fe, Co, Ni, Zn, Mg compounds are known^10^), we hypothesize that the unidentified phase maintains the La_2_Ti_2_O_7_ stoichiometry, but the deposition conditions have not provided enough kinetic energy to sufficiently order the oxygen planes as well-defined layers between every four perovskoite slabs. Instead, partially complete oxygen planes have formed between every two perovskite slabs, and this appears as the *A*_2_*B*_2_O_8_ structure in TEM (which is insensitive to precise oxygen stoichiometry). The crystal structure of the La-Ti-O system does not necessarily directly correlate to the oxygen stoichiometry^10^, and thus we speculate that the La_2_Ti_2_O_7_ stoichiometry can exist in both the *A*_2_*B*_2_O_8_ and the *AB*O_3_ crystal structures with disordered oxygen vacancies or excess oxygen dopants to maintain charge neutrality.

### LTO on STO(001)

Due to the ~4.5° tilt between the STO[001] and LTO[021] directions when LTO is deposited on STO(001), a single high-resolution θ−2θ XRD scan will not capture both film and substrate peaks^16^. Fig. 1(b) shows the out-of-plane XRD patterns when the diffractometer is aligned to the substrate (curve (i)), and when it is aligned to the diffraction feature at ~41° 2θ (curve (ii)). In curve (ii), the diffraction peaks corresponding to the STO(001) substrate do not appear, but three diffraction features remain. Two of these peaks can be indexed to LTO(021) (2θ = 21.135°) and LTO(042) (2θ = 43.034°), which is the expected orientation for epitaxial LTO on STO(001)^16^. The diffraction feature at ~47.7–48.1° 2θ cannot be indexed to an LTO plane in the 〈021〉 family^11^. As revealed by the reciprocal space map (RSM) plotted in Fig. 1(c), this diffraction feature exhibits the same alignment with the STO substrate as does the LTO(420) reflection, and thus corresponds to an epitaxial phase. Three lobes from each feature are present in the RSM, corresponding to planes aligned parallel to the STO(001) surface (intensity at 0° from the surface normal), and planes tilted ±~4° away from the surface normal. An additional diffraction feature between the LTO(021) and STO(001) peaks (~22° 2θ) is present when aligned to the substrate, but does not appear when aligned to the LTO film. This peak arises from a secondary phase of perovskite LaTiO_3_(001), which is well aligned to the STO(001) substrate. After annealing at 1100 °C for 4 h in air, little change is observed in the diffraction features (curves (iii) and (iv)).

The primary epitaxial orientation, LTO(021)//STO(001), is confirmed by the STEM-HAADF images in Fig. 3. In these images, the LTO(001) direction lies approximately 45° to the substrate, and thus the additional oxygen planes also lie along this direction. On the STO(001) surface, which possesses a cubic surface net of atoms, there are four equivalent possible orientations (separated by 90° around the STO(001) surface normal) for the LTO(021) film to nucleate and grow. In Fig. 3, twinned left and right orientations of LTO[001], separated by 180°, are shown. The other two equivalent orientations (LTO[001] pointed out of the image and into the image) are likely also present, but are difficult to distinguish; in these orientations, the atomic columns of the PLS structure are not highly aligned, and thus these orientations would appear weakly cubic but fairly disordered in the STEM images. Disordered regions are observed in Fig. 3(a) which might correspond to these orientations. However, the cubic atomic structure observed in portions of these regions may also arise from perovskite LaTiO_3_ secondary phases, since LaTiO_3_(001) was also observed in the XRD patterns (Fig. 1(b)).

**Figure 4:**
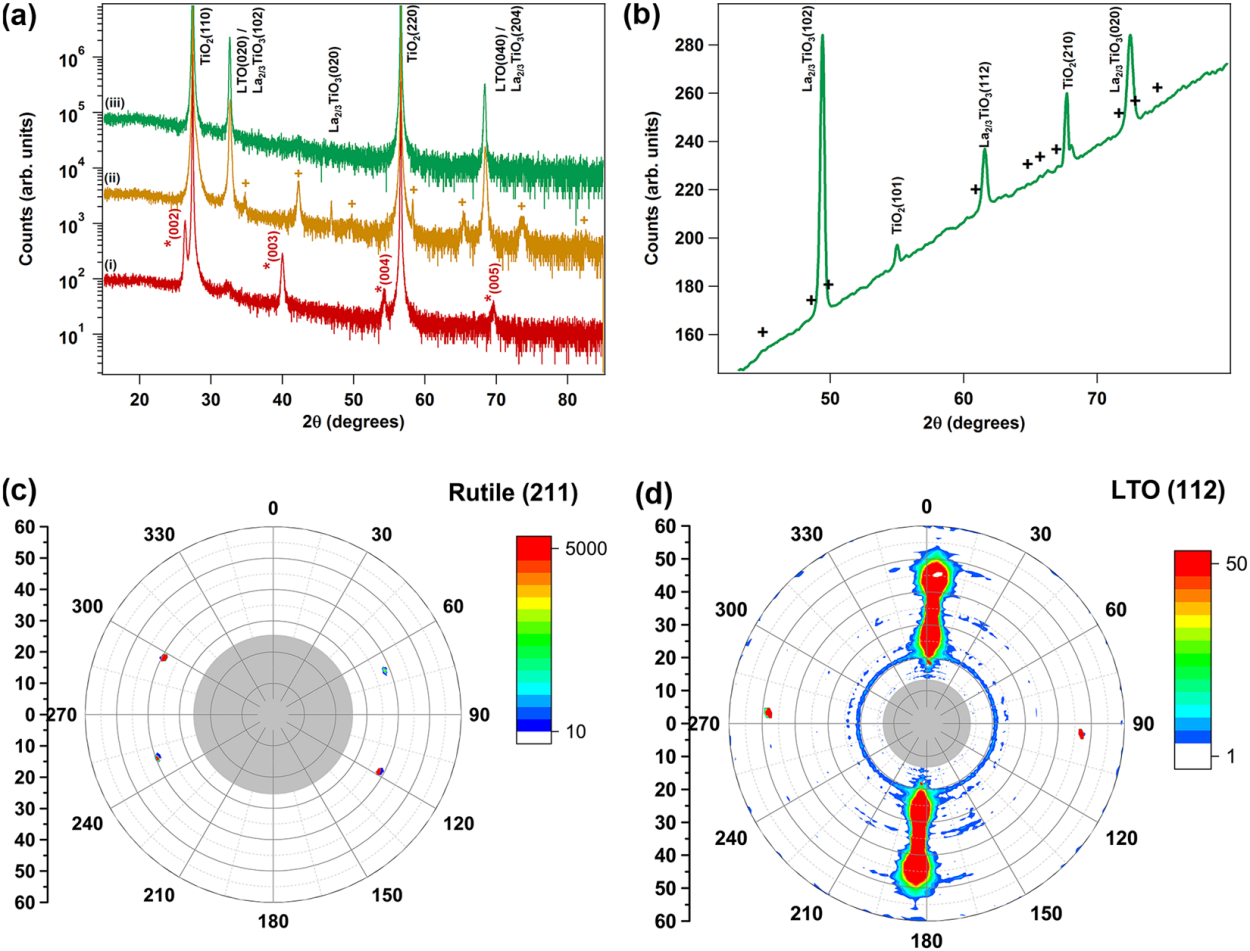
XRD patterns of La_2_Ti_2_O_7_ deposited on rutile TiO_2_(110) at 925–950 °C. (**a**) Curve (i) as-deposited with unidentified phase marked with *; (ii) film from (i) after annealing 1000 °C/8 h in air, with the 〈00l〉 orientation of La_2_Ti_2_O_7_ marked with +; (iii) Film deposited on Nb-doped TiO_2_(110) and annealed at 1000 °C/8 hrs in air. (**b**) μ−XRD powder scan of film (iii), confirming the presence of La_2/3_TiO_3_. Positions of expected La_2_Ti_2_O_7_ peaks are marked with +. (**c**) Rutile(211) pole figure. (**d**) La_2/3_TiO_3_(112) pole figure. Pole figures collected with μ-XRD; gray shading in the center of the figures indicates the region where no data was collected.

### LTO on TiO2(110)

To orient the film such that the direction of ferroelectric coupling (the LTO[010] direction) is pointed out of the plane of the film, LTO depositions were attempted on rutile TiO_2_(110). There exists a reasonable lattice match of 1.3% between the Ti spacing along the TiO_2_[$1\overline{1}0$] direction (*d* = 6.496 Å) and the average La spacing along the LTO[100] direction (*d*_average_ = 6.58 Å). The lattice match in the perpendicular in-plane direction is not as favorable: the Ti spacing in the TiO_2_[001] direction is 2.958 Å, while the average spacing between La cations in the LTO[001] direction is 3.92 Å, resulting in a lattice misfit of 32.5%. However, there exists a coincident lattice match between LTO and TiO_2_ in this direction, with four TiO_2_ units (11.832 Å) nearly equal to three LTO units (11.76 Å). Combined with the favorable lattice match in the LTO[100] direction, epitaxy may be possible. As shown in curve (i) of Fig. 4(a), deposition on TiO_2_(110) does not result in an LTO film with the (010) orientation. Instead, surprisingly, the same *A*_2_*B*_2_O_8_-like phase as observed in depositions on STO(110) results, with the same [001] out-of-plane orientation. As with LTO/STO(110), annealing the LTO/TiO_2_(110) film at 1000 °C for 8 h in air transforms this phase (Fig. 4(a), curve (ii)). However, in contrast to the deposition on STO(110), the dominant orientation on TiO_2_(110) appears to be LTO(010); this is the expected orientation from the lattice matching argument above. A weaker set of diffraction peaks corresponding to a secondary epitaxial orientation of LTO(001) is also observed. Interestingly, deposition of a thicker (1000 Å) LTO film on Nb-doped TiO_2_(110), annealed at 1000 °C in air for 8 h, results in an XRD pattern (curve (iii)) which is free of secondary orientations, and appears to consist only of LTO 〈0*l*0〉 peaks. However, glancing-angle μXRD shown in Fig. 4(b) reveals that the dominant crystal structure matches best to La_2/3_TiO_3_, an orthorhombic perovskite phase with ordered A-site vacancies^21,22^, not La_2_Ti_2_O_7_. The orthorhombic (102) plane (pseudocubic (101)) of La_2/3_TiO_3_ is oriented parallel to the TiO_2_(110) substrate. As revealed by pole figures of the Nb:TiO_2_(211) and La_2/3_TiO_3_(112) reflections in Fig. 4(c), the orthorhombic phase exhibits in-plane as well as out-of-plane epitaxial relationships to the Nb:TiO_2_ substrate. The correspondence of the La_2/3_TiO_3_(102) reflection position to the “LTO(020)” reflection in Fig. 2(a) curve (ii) confirms that the oriented phase in this film is also La_2/3_TiO_3_ (note that the assignment of the PLS LTO(001) orientation doesn’t change).

**Figure 5:**
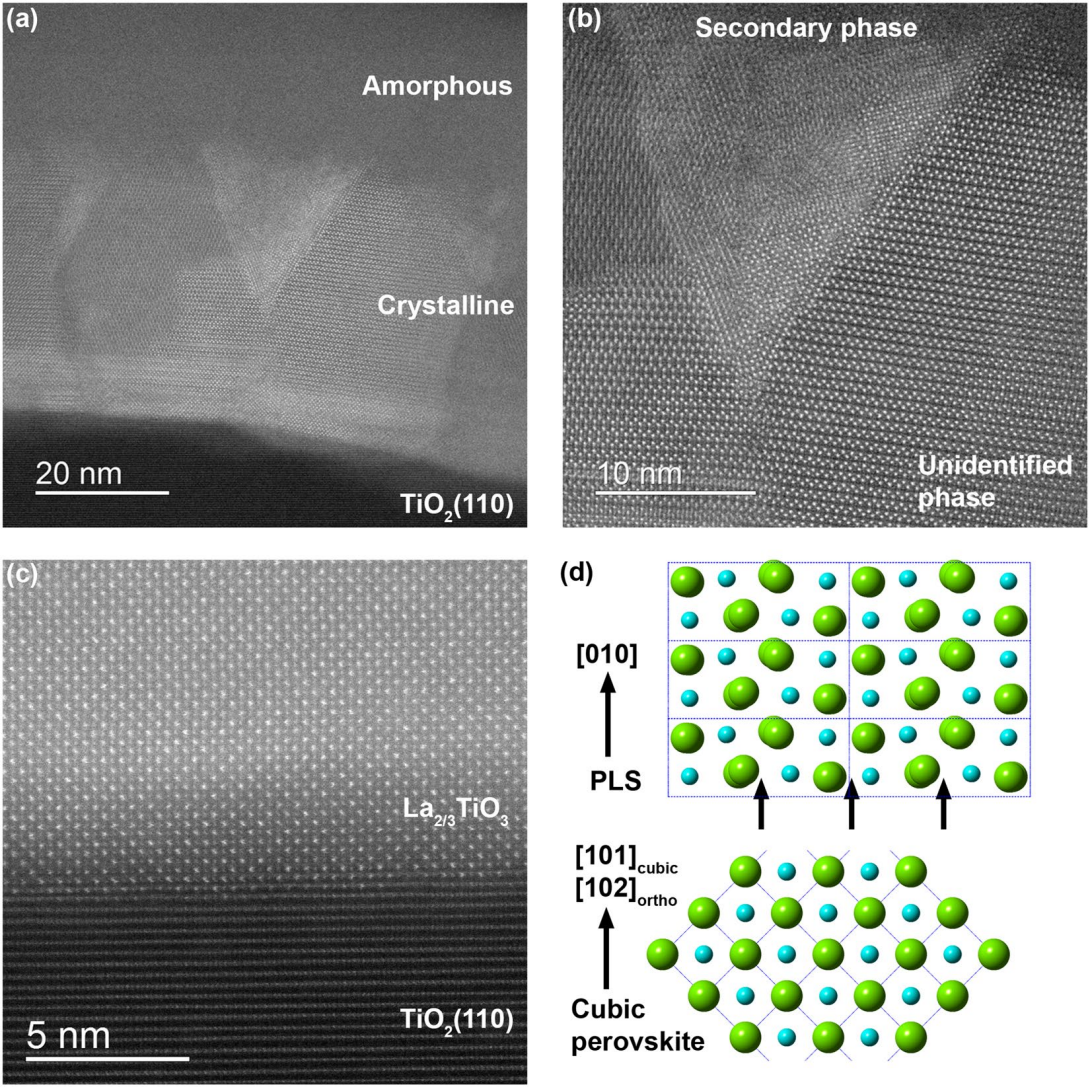
STEM-HAADF images of LTO/TiO_2_(110). (**a**), (**b**) As-deposited film. Regions of amorphous LTO, triangular defects of a secondary phase, and an unidentified phase similar to that observed for LTO/STO(110) are indicated. (**c**) STEM-HAADF image of LTO/TiO_2_(110) after annealing at 1000 °C/8 h in air. The LTO phase is La_2/3_TiO_3_(102). (**d**) Sketch of the correspondence of cation positions in the PLS [010] and cubic/pseudo-cubic perovskite [101] (orthorhombic [102]) directions.

The STEM-HAADF images presented in Fig. 5 provide more insight into the structure of La_2/3_TiO_3_ and LTO on TiO_2_(110). As shown in Fig. 5(a), the as-deposited LTO film (curve (ii) in Fig. 2(a)) is crystalline at the TiO_2_(110) interface and extending approximately halfway through the film thickness, but the top portion of the film (~30 nm) is amorphous. This amorphous phase may arise from excess La that has been expelled from the La_2/3_TiO_3_ crystal structure. The same unidentified cubic phase that is present in LTO/STO(110) depositions (Fig. 2(b)) can be clearly observed on the right side of Fig. 5(b). On the left side of the image are regions which we interpret as in-plane rotational domains of this same structure. In the center of the image is a triangular defect of another phase. Similar triangular defects were observed in other regions of the film. There appears to be a specific orientation relationship between the two phases at their interface, but identification of either phase is non-trivial and beyond the scope of this paper.

After annealing at 1000 °C for 8 h, the film thickness becomes highly non-uniform (not shown). Despite the annealing treatment, which was sufficient to promote this bulk transfer of material, the top portion of the film remains amorphous. The thickness of the amorphous region has decreased (~16 nm), and this thickness remains fairly uniform regardless of the variation in the overall film thickness. In the high-resolution lattice image presented in Fig. 5(c), the LTO lattice has transformed from the unidentified phases shown in Fig. 5(b); however, the lattice image does not exhibit the same pattern as the PLS lattice images in Figs 2(d) and 3(c). This provides another indication that the predominant phase is La_2/3_TiO_3_, not La_2_Ti_2_O_7_. Schematic diagrams of both the La_2/3_TiO_3_ atomic positions in a multi-unit-cell slab of the cubic perovskite structure (for simplicity, a cubic unit cell, not the orthorhombic doubled cell^21^, is shown) and the LTO atomic positions in a six-unit-cell slab of the PLS structure are sketched in Fig. 5(d). The correlation between the schematic atomic positions and the lattice positions in the image confirms that the epitaxial phase is La_2/3_TiO_3_ with the orthorhombic [102] direction perpendicular to the interface. In this crystalline orientation, the superstructure contrast arising from the ordered *A*-site vacancies is not visible, in contrast to STEM images of the [100] projection^23^. Inspection of the lattice schematics confirms that the orthorhombic La_2/3_TiO_3_[102] direction corresponds closely to the LTO[010] direction. The successful epitaxial growth of La_2/3_TiO_3_(102) on TiO_2_(110) indicates that further optimization of the deposition conditions could realize the epitaxial LTO[010] phase.

### Piezoelectric properties of LTO thin films

**Figure 6:**
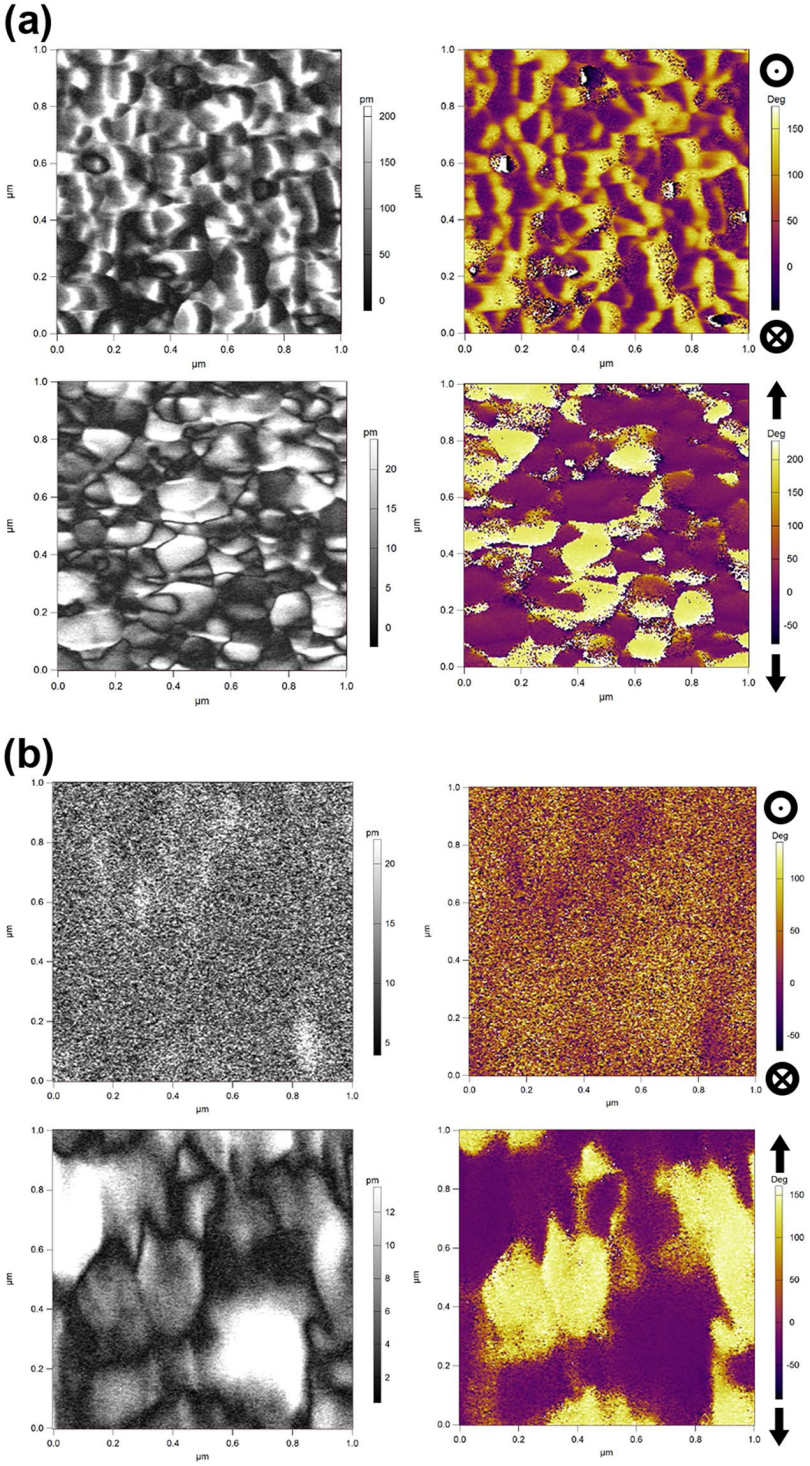
PFM vertical amplitude (top left) and out-of-plane phase (top right), and lateral amplitude (bottom left) and in-plane phase (bottom right) of LTO films deposited on (**a**) Nb:STO(001) and (**b**) Nb:STO(110). A phase value of 180° indicates polarization out of the plane of the image (top right images) or vertically in the plane of the image (bottom right images).

PFM was employed to characterize the piezoelectric properties of LTO both in and out of the film plane for thick films (100 nm) deposited on Nb:STO(110), Nb:STO(001), and Nb:TiO_2_(110). The amplitude and phase results for LTO films deposited on Nb:STO(001) and Nb:STO(110) are presented in Fig. 6(a) and (b), respectively. The piezoelectric coupling direction in the PLS structure of LTO is parallel to the additional oxygen planes, along the LTO[010] direction. From the XRD and STEM data above, LTO/STO(001) possesses this direction at an approximately 45° angle to the substrate surface. PFM measurements in Fig. 6(a) confirm that clear piezoelectric coupling is observed for LTO/Nb:STO(001). The piezoresponse exhibits similar magnitude and spatial structure in-plane and out-of-plane, consistent with a 45° piezoelectric coupling direction with components of similar magnitude in-plane and out-of-plane. A comparison of the top two images in Fig. 6(a) indicates that each film grain in the amplitude image (left) consists of two, opposing polarization directions separated by a domain wall (seen as a color change, indicating a phase reversal, in the right-hand image). In the plane of the film (bottom images), the grains each consist of a single polarization domain.

In contrast, no structure indicative of a polarization reversal is observed in the out-of-plane images for LTO/Nb:STO(110), as seen in Fig. 6(b) (top images). This is expected for LTO deposited on STO(110) with the piezoelectric LTO[001] direction lying entirely in the film plane, with no out-of-plane component. Structure is observed in the lateral amplitude and in-plane phase images (Fig. 6(b), bottom), confirming an in-plane piezoelectric orientation. As with LTO/STO(001), the film grains each consist of a single in-plane piezoelectric domain, with domain walls between grains.

**Figure 7:**
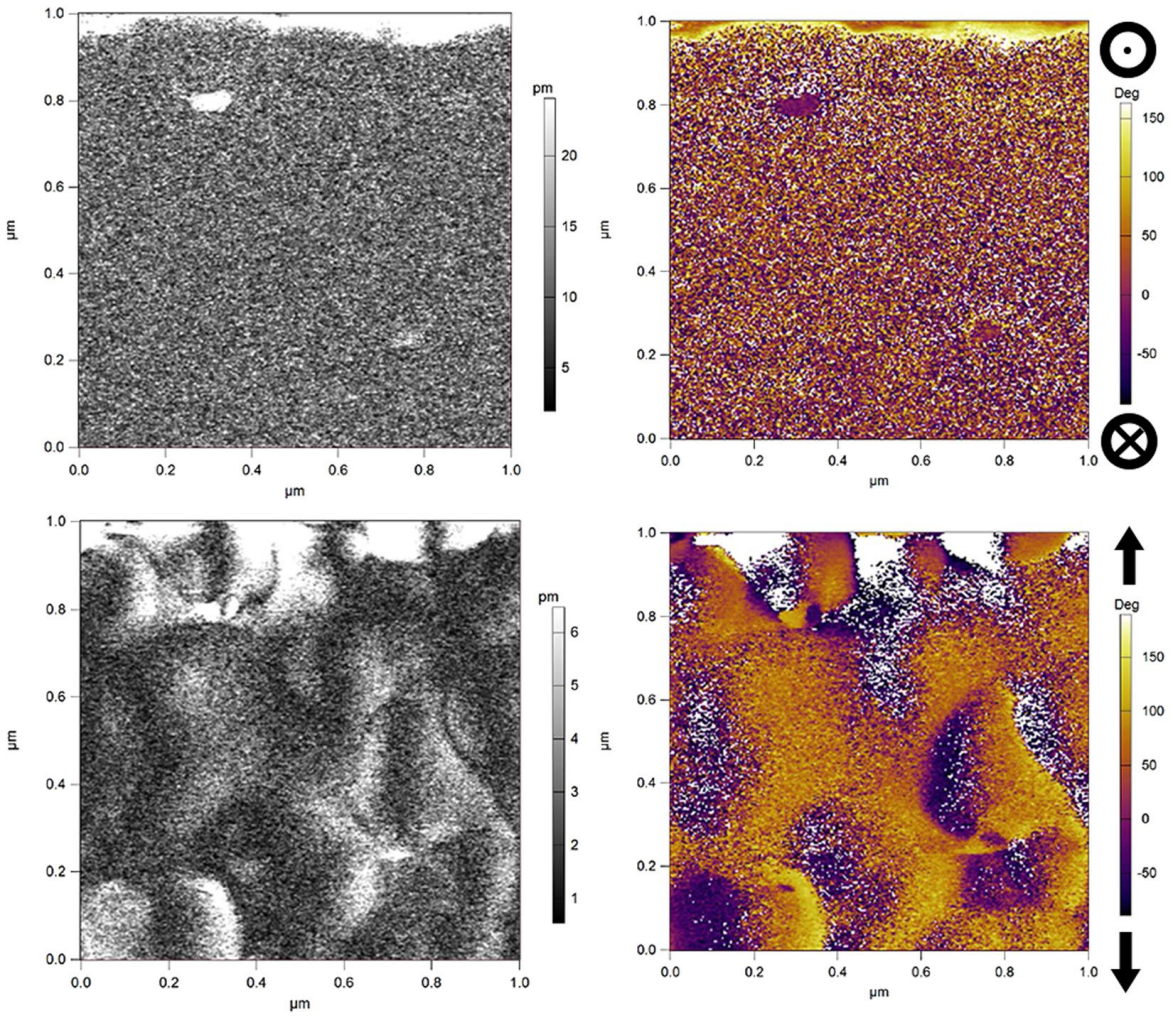
PFM vertical amplitude (top left) and out-of-plane phase (top right), and lateral amplitude (bottom left) and in-plane phase (bottom right) of La_2/3_TiO_3_ film deposited on Nb:TiO_2_(110). A phase value of 180° indicates polarization out of the plane of the image (top right image) or vertically in the plane of the image (bottom right image).

A strong out-of-plane piezoelectric response is expected for LTO/Nb:TiO_2_(110) if the piezoelectric LTO[010] direction is oriented out-of-plane. Instead, the dominant phase is epitaxial La_2/3_TiO_3_, an orthorhombic perovskite structure which is not piezoelectric. In Fig. 7, as expected, no strong piezoelectric signals are observed either out-of-plane or in-plane.

Quantitative values of the piezoelectric coefficient (d_33_) cannot be obtained by our PFM measurements. However, the strength of piezoelectric coupling can be qualitatively evaluated by comparing the maximum vertical PFM amplitude, normalized to the applied voltage, for each film. These values are 22, 2.2, and 1.7 pm/V for LTO films on STO(001), STO(110), and TiO_2_(110), respectively. Clearly, the LTO film on STO(001) shows an enhanced piezoelectric response compared to the films with other crystalline orientations. Epitaxial deposition of LTO[021] on STO(001) would be preferred for thin film piezoelectric devices. In addition, further optimization of the LTO deposition parameters to stabilize epitaxial LTO[010] on TiO_2_(110) (or another suitable substrate) is expected to promote even stronger out-of-plane piezoelectric coupling.

While La_2/3_TiO_3_ may not be of interest as a piezoelectric material, it has been explored as a microwave dielectric with promising properties^23–25^. La_2/3_TiO_3_ is also the parent compound of ionic^22^ and lithium ion^26^ conductors. The orthorhombic structure of La_2/3_TiO_3_, with ordered A-site cation vacancies, is difficult to stabilize in bulk form without the addition of dopant cations^23,25^. Epitaxial stabilization of undoped La_2/3_TiO_3_ on TiO_2_(110) is a promising route to realize pure La_2/3_TiO_3_ for both fundamental studies and device applications.

In summary, epitaxial thin films of the high-temperature piezoelectric material La_2_Ti_2_O_7_ were deposited on STO(110), STO(001), and rutile TiO_2_(110) substrates by pulsed laser deposition. Reasonable control of the film orientation can be achieved when depositing LTO on substrates with various orientations, as confirmed by XRD patterns and STEM measurements. PFM measurements confirmed that LTO/STO(001) possesses strong piezoelectric coupling in a direction approximately 45° to the film plane; both in-plane and out-of-plane piezoelectric signals were observed. In the out-of-plane direction, each film grain consists of two domains with opposite polarization direction; in-plane, only one polarization direction is observed for each grain. For LTO films deposited on STO(110), only in-plane polarization is observed, as expected. The case of LTO on TiO_2_(110) is more complex. The film structure consists of several phases and orientations, and the dominant orientation is epitaxial La_2/3_TiO_3_(102). This phase is not piezoelectric, but is of interest as a microwave dielectric material and an ion conductor. The results presented here confirm that the strength of piezoelectric coupling can be enhanced in epitaxial LTO thin films, which may be of interest for high-temperature sensors and piezoelectric devices.

## Methods

### Film synthesis

Thin films of LTO were deposited from a stoichiometric La_2_Ti_2_O_7_ target by PLD (248 nm wavelength KrF laser, fluence ~2 J/cm^2^, repetition rate 1 Hz). Substrates were held at a temperature of 900–950 °C in 0.5–25 mTorr O_2_ during the deposition. Single crystal STO(001), Nb:STO(001), STO(110), Nb:STO(110), TiO_2_(110), and Nb:TiO_2_(110) substrates were utilized; in all cases, substrates were degreased before loading into the deposition chamber. LTO films for x-ray diffraction (XRD) and scanning transmission electron microscopy (STEM) analysis were nominally 500 Å thick, while those deposited on Nb-doped conducting substrates for piezoresponse force microscopy (PFM) were nominally 1000 Å thick.

### Crystalline properties

High-resolution XRD patterns were collected on a Philips X’Pert Materials Research Diffractometer (MRD) using Cu K_α1_ radiation monochromated with a hybrid mirror/4 crystal monochromator and fixed-slit detector optics. A Rigaku D/MAX RAPID II microdiffractometer with a curved imaging plate and a rotating Cr anode (Cr Kα = 2.2897 Å) operating at 35 kV and 25 mA was used to collect microbeam XRD (μXRD) and pole figure patterns. Cross-sectional STEM samples were fabricated using a focused ion beam (FIB) lift out technique with a FEI Helios microscope operating at 0.5–30 keV ion beam energy. High-angle annular dark field (STEM-HAADF) images were acquired using a JEOL ARM-200CF microscope operating at 200 keV with a 27.5 mrad convergence and 82.6 mrad inner collection semi-angles, respectively. Additional STEM-HAADF images were acquired using an FEI Titan 80–300 microscope operating at 300 keV.

### Piezoelectric properties

Piezoresponse force microscopy (PFM) was performed using a commercially available atomic force microscope (AFM) (MFP-3D, Asylum Research) and Pt coated AFM tips (EFM-50, Nanoworld) at ac modulation voltage of 2 V_pp_ (peak to peak) and scan frequency of 1 Hz. The vertical and lateral PFM images were obtained with drive frequencies between 395.61 and 410.19 kHz, and between 659.09 and 743.98 kHz, respectively, near the contact resonance for vertical and torsional motions of PFM cantilevers.

### Data availability

All data generated or analysed during this study are included in this published article.
